# The association between objective cognitive measures and ecological-functional outcomes in COVID-19

**DOI:** 10.3389/fpsyg.2022.903697

**Published:** 2022-11-01

**Authors:** Marcella Ottonello, Elena Fiabane, Edoardo Nicolò Aiello, Marina Rita Manera, Francesca Spada, Caterina Pistarini

**Affiliations:** ^1^Department of Physical and Rehabilitation Medicine of Genova Nervi Institute, Istituti Clinici Scientifici Maugeri IRCCS, Genova, Italy; ^2^PhD Program in Neuroscience, School of Medicine and Surgery, University of Milano-Bicocca, Monza, Italy; ^3^Psychology Unit of Pavia Institute, Istituti Clinici Scientifici Maugeri IRCCS, Pavia, Italy; ^4^Department of Neurorehabilitation of Pavia Institute, Istituti Clinici Scientifici Maugeri IRCCS, Pavia, Italy

**Keywords:** SARS-CoV-2, COVID-19, functional independence measures, cognitive screening, neuropsychology, ecological validity

## Abstract

**Background:**

Cognitive dysfunctions, both subjective and detectable at psychometric testing, may follow SARS-CoV-2 infection. However, the ecological-functional relevance of such objective deficits is currently under-investigated. This study thus aimed at investigating the association between objective cognitive measures and both physical and cognitive, ecological-functional outcomes in *post-*COVID-19.

**Methods:**

Forty-two COVID-19-recovered individuals were administered the Mini-Mental State Examination (MMSE) and the Montreal Cognitive Assessment (MoCA). The Functional Independence Measure (FIM) was adopted to assess functional-ecological, motor/physical (FIM-Motor) and cognitive (FIM-Cognitive) outcomes at admission (T0) and discharge (T1).

**Results:**

When predicting both T0/T1 FIM-total and-Motor scores based on MMSE/MoCA scores, premorbid risk for cognitive decline (RCD) and disease-related features, no model yielded a significant fit. However, the MoCA - but not the MMSE significantly predicted T0/T1 FIM-Cognitive scores. The MoCA was significantly related only to T0/T1 FIM-Cognitive Memory items.

**Discussion:**

Cognitive measures are not associated with physical/motor everyday-life outcomes in *post*-COVID-19 patients. The MoCA may provide an ecological estimate of cognitive functioning in this population.

## Introduction

SARS-CoV-2 infection may affect cortical/sub-cortical structures through both direct (neurotropism) and indirect (e.g., neuroinflammation, iatrogenic effects of treatments) pathways and thus entail both subjective and objective cognitive dysfunctions within the dysexecutive/inattentive and amnesic *spectrum* (Daroische et al., 2021). Since a detrimental influence of COVID-19-related cognitive aftermaths on prognosis has been postulated (Evans et al., 2021), cognitive screening is recommended in this population (Daroische et al., 2021; Manera et al., 2022). Moreover, it has been highlighted that the pandemic itself might indirectly affect cognition as representing, at a psychosocial level, a significant stressor (Santangelo et al., 2021; Scuotto et al., 2021).

However, the actual interplay between objective cognitive sequelae and ecological-functional outcomes – herewith intended as both physical independence in daily living and the ability to efficiently and effectively engage one’s cognitive functions to cope with everyday-life activities – has been to date scarcely explored in this population, and remains incompletely understood (Daroische et al., 2021; Carda et al., 2020; Evans et al., 2021; Manera et al., 2022), with evidence suggesting the independence of these two clusters (Carda et al., 2020).

To explore the interplay between cognitive dysfunctions and ecological-functional outcomes in *post-*COVID-19 patients would be thereupon relevant in order to determine the extent to which such sequelae impact on the lives of individuals recovered from the disease, this informing on their prognosis and, in turn, on the need for implementing rehabilitative interventions (Carda et al., 2020; Wade, 2020), especially in the light of the growing demand of multi-disciplinary programs aimed at managing the aftermaths of SARS-CoV-2 infection (Ahmad et al., 2022; Fugazzaro et al., 2022), including cognitive ones (Rolin et al., 2022).

This study thus aimed at investigating the association between objective cognitive measures and both physical and cognitive, ecological-functional outcomes in a mono-centric cohort of *post*-COVID-19 inpatients by also accounting for premorbid and disease-related features possibly affecting cognition.

## Materials and methods

### Materials

Data from *N* = 42 *post*-COVID-19 patients referred to the Department of Physical and Rehabilitation Medicine of Istituti Clinici Scientifici Maugeri of Pavia (Northern Italy) between 2020 and 2021 were retrospectively collected (Table 1). Disease severity was codified as “asymptomatic,” “mildly symptomatic,” “mild-to-moderate” (requiring O_2_ but not ventilation) and “moderate-to-severe” (requiring either non-invasive ventilation or ICU). Patients were classified as previously-at-risk for cognitive decline (RCD+) or not (RCD−) based on remote, recent and COVID-19-related neurological/psychiatric history (Manera et al., 2022; Aiello et al., 2022a,b,c). Cognition was assessed *via* the Mini-Mental State Examination (MMSE; Carpinelli Mazzi et al., 2020) and the Montreal Cognitive Assessment (MoCA; Aiello et al., 2022d), whereas functional-ecological outcomes *via* the Functional Independence Measure (FIM; Linacre et al., 1994). The FIM is an 18-item, clinician-report tool assessing both motor (FIM-Motor) and cognitive (FIM-Cognitive) functioning in an ecological fashion. The FIM-Motor assesses a wide range of physical-motor functions and activities of daily living – i.e. the level of independence in self-care, the extent to which a patient is able to perform movements without assistance and the degree of sphincteric control. The FIM-Cognitive subscale instead encompasses 5 items: Comprehension, Expression, Social interaction, Problem solving and Memory, all being operationalized through items that ecologically target cognitive efficiency and effectiveness within everyday-life activities. While the first items two focus on patients’ language functioning within both the receptive and productive modality, and thus communicative abilities in daily living, the last three sample one’s capability to adequately engage executive and memory skills in everyday-life tasks.

**Table 1:** Patients’ background and clinical measures.

Outcome		Admission	Discharge	*p*
N		42	-	-
Age (years)		68.83 ± 10.1 (46–85)	-	-
Sex (male/female)		27/15	-	-
Education (years)		11.24 ± 3.82 (3–18)	-	-
Disease duration (days)		40.1 ± 27.29 (2–113)	-	-
Time from onset (days)		66.19 ± 33.38 (7–173)	-	-
Severity (%)				
	Asymptomatic	9.5%	-	-
	Mildly symptomatic	7.1%	-	-
	Mild-to-moderate	23.8%	-	-
	Moderate-to-severe	59.5%	-	-
ICU		47.6%	-	-
Steroids		28.6%	-	-
Risk for cognitive decline (%)		28.6%	-	-
Mini-Mental State Examination		26.55 ± 2.72 (19–30)	-	-
Montreal Cognitive Assessment		20.45 ± 3.43 (12–25)	-	-
Functional Independence Measure				
	Total	78.98 ± 16.33 (49–111)	109.31 ± 12.75 (82–127)	<0.001*
	Motor	46.17 ± 15.18 (17–76)	75.76 ± 11.21 (55–92)	<0.001*
	Cognitive	32.81 ± 3.52 (20–35)	33.55 ± 2.88 (23–35)	<0.001*
	Comprehension	6.71 ± 0.67 (4–7)	6.81 ± 0.51 (5–7)	0.102^§^
	Expression	6.71 ± 0.6 (4–7)	6.88 ± 0.4 (5–7)	0.008^§^
	Social Interaction	6.64 ± 0.73 (4–7)	6.74 ± 0.59 (5–7)	0.046^§^
	Problem solving	6.33 ± 1.1 (3–7)	6.55 ± 0.89 (3–7)	0.007^§^
	Memory	6.4 ± 1.04 (3–7)	6.57 ± 0.91 (3–7)	0.008^§^

ICU = intensive care unit.

* t-Statistics.

§ Wilcoxon’s W-statistics.

The FIM was administered 3 days after admission (T0) and 3 days before discharge (T1); cognitive assessment was performed within the first week of hospitalization (average inpatient time being 1 month).

### Statistics

The minimum sample size was estimated through G*Power 3.1.9.4 at *N* = 33 based on a non-parametric correlational model with ρ = 0.45, two-tailed α = 0.05 and 1-β = 0.8. Based on homoscedasticity and normality assumptions being met, either linear model analyses or non-parametric approaches were carried out. Consistently, linear regression analyses were run on FIM-total and FIM-Cognitive/-Motor scales, whereas Spearman’s correlations were run when dealing with FIM-Cognitive items. Each regression model encompassed the following predictors, which were entered simultaneously: global cognition (MMSE/MoCA), sex, disease severity, steroidal treatment (yes/no), ICU admission (yes/no), RCD (+/−) and disease duration (in days). In order to avoid collinearity and to comparatively test the MMSE vs. the MoCA in predicting FIM outcomes, such models were implemented separately for the two screeners. Collinearity between predictors was checked for by addressing the variance inflation factor (VIF) and tolerance index (TI), which were judged as abnormal if >10 and < 0.1, respectively (Dormann et al., 2013). MMSE and MoCA scores were adjusted for age and education according to current norms (Carpinelli Mazzi et al., 2020; Aiello et al., 2022d). SPSS 27 (IBM Corp., 2020) was used to analyze data; α was set at 0.005.

## Results

Background and clinical measures are summarized in Table 1. Prevalence of below-cut-off scores was 19% on the MMSE and 28.6% on the MoCA. MMSE and MoCA adjusted scores were moderately associated [*r_s_*(42) = 0.44; *p* = 0.004], this supporting the present choice to implement separate models for the two screeners.

When predicting both T0 and T1 FIM-total, FIM-Motor and FIM-Cognitive scales based on MMSE scores, sex, disease severity, steroidal treatment, ICU admission, RCD and disease duration, no model yielded a significant fit [0.17 *≤ R*^2^ *≤ 0.*28; 0.84 *≤ F*(7,34) *≤* 1.87*; p ≥ 0*.106]. The same models similarly revealed no significant fits when addressing the MoCA instead of the MMSE as predictor and FIM-total and FIM-Motor T0/T1 scores as outcomes [0.12 *≤ R*^2^ *≤ 0.*26; 0.56 *≤ F*(7,34) *≤* 1.67*; p ≥ 0*.15].

However, the MoCA proved to be the only significant predictor on both T0 (*β* = 0.46; *t =* 3.11; *p* = 0.004) and T1 (*β* = 0.44; *t =* 2.77; *p* = 0.009) FIM-Cognitive scores - other independent variables yielding non-significant effects (|0.02| *≤ β ≤* |0.26|; |0.02| *≤ t ≤* |1.48|*; p ≥ 0*.147). In order to further explore the association between FIM-Cognitive items and MoCA scores, Spearman’s correlation were run, revealing that the MoCA was significantly associated only with the Memory item at both T0 [*r_s_*(42) = 0.41; *p* = 0.007) and T1 (*r_s_*(42) = 0.41; *p* = 0.007] - other coefficients being non-significant (0.15 *≤ r_s_ ≤ 0.*3; *p ≥ 0*.053).

Collinearity statistics for the entered predictors fell under the normal ranges as to both the models addressing the MMSE (VIF ≤ 2.65; TI ≥ 0.38) and the MoCA (VIF ≤ 2.63; TI ≥ 0.38).

## Discussion

The present findings suggest that cognitive measures are not associated with physical/motor everyday-life outcomes in *post*-COVID-19 patients – at variance with objective and ecological measures of cognition, which in fact appeared to be related. Notably, such an association proved to be measure-dependent – as the MoCA, but not the MMSE, predicted FIM-Cognitive scores. Relevantly, the MoCA proved to be able to predict the FIM-Cognitive at both T0 and T1, this suggesting that it can be addressed as a valid measure to track ecological cognitive functioning over the course of a rehabilitation program.

This finding is in line with previous contributions suggesting that the MoCA is abler than the MMSE in detecting cognitive sequelae of SARS-CoV-2 infections (Aiello et al., 2022b; Biagianti et al., 2022). Hence, not only the MoCA appears to be suitable for assessing cognition in *post*-COVID-19 patients, but may also provide an ecological estimate of their cognitive functioning.

In this respect, it is noteworthy that the only FIM-Cognitive item found to be significantly associated with MoCA scores was the one assessing memory functioning; this finding is indeed in line with memory complaints/deficits being highly reported/detected in COVID-19-recovered individuals (Søraas et al., 2021; Aiello et al., 2022a,c). In this respect, it should be however noted that this finding might be biased by the fact that the MoCA relevantly loads on memory functions. Therefore, further research is needed that address the correlation between such a screener and ecological measures of cognition that encompass several functions/domains, beyond those addressed by the FIM.

A caveat should be nonetheless listed as to the association between ecological and objective measures of cognition, namely that, at least at a group level, FIM-Cognitive item scores were not suggestive of a clinically meaningful prevalence of cognitive impairment in the present sample, at variance with MMSE and MoCA scores. Such a discrepancy might be explained by the fact that these two approaches to cognitive measurement are intrinsically different – the FIM being clinician-report and based on clinical observations, the MMSE and MoCA being performance-based tests –, as well as that the FIM-Cognitive and the MMSE/MoCA do not completely overlap as to their target constructs.

A further limitation of this work lies in the fact that behavioural and psychiatric features, which are acknowledged to possibly occur as a consequence of SARS-CoV-2 infection (Schou et al., 2021), were not herewith addressed – in spite of their relevance towards the ecological-functional outcome of patients with supposed brain disorders (Aiello et al., 2022e). Future studies are therefore advisable that more comprehensively assess functional outcomes in COVID-19-recovered individuals by also including behavioural and psychiatric measures.

Finally, findings herewith reported appear to be sound due to the fact that several disease-related and premorbid confounders were covaried when assessing the association between cognition and functional-ecological measures. Thereupon, objective, specific psychometric estimates of global cognitive efficiency provided by the MoCA are likely to yield an actual picture of ecological cognitive functioning in this population, this prompting the adoption of this screener as a valid cognitive outcome when referring to *post*-COVID-19 patients within both clinical and research settings.

## Data availability statement

The raw data supporting the conclusions of this article will be made available by the authors, without undue reservation.

## Ethics statement

This study received approval by Istituti Clinici Scientifici Maugeri SpA SB (I.D.: 2470, 8 September 2020). The patients/participants provided their written informed consent to participate in this study.

## Author contributions

MO and EF: conceptualization, resources, data collection, and revision. EA: statistical analyses, drafting, and revision. MM: data collection, resources, and revision. FS: data collection and revision. CP: resources and revision. All authors contributed to the article and approved the submitted version.

## Funding

The open access fees were funded by IRCCS Istituti Clinici Scientifici Maugeri.

## Conflict of interest

The authors declare that the research was conducted in the absence of any commercial or financial relationships that could be construed as a potential conflict of interest.

## Publisher’s note

All claims expressed in this article are solely those of the authors and do not necessarily represent those of their affiliated organizations, or those of the publisher, the editors and the reviewers. Any product that may be evaluated in this article, or claim that may be made by its manufacturer, is not guaranteed or endorsed by the publisher.
